# High quality draft genome sequence and description of *Occidentia massiliensis* gen. nov., sp. nov., a new member of the family *Rickettsiaceae*

**DOI:** 10.1186/1944-3277-9-9

**Published:** 2014-12-08

**Authors:** Oleg Mediannikov, Thi-Thien Nguyen, Lesley Bell-Sakyi, Roshan Padmanabhan, Pierre-Edouard Fournier, Didier Raoult

**Affiliations:** 1 URMITE, Aix-Marseille Université, Marseille, France; 2 URMITE, Campus commun UCAD-IRD d'Hann, Dakar, Senegal; 3 The Tick Cell Biobank, The Pirbright Institute, Surrey, UK; 4 Special Infectious Agents Unit, King Fahd Medical Research Center, King Abdulaziz University, Jeddah, Saudi Arabia

**Keywords:** *Occidentia massiliensis*, Genome, Senegal, Soft tick, *Ornithodoros sonrai*, Taxonogenomics

## Abstract

The family *Rickettsiaceae* currently includes two genera: *Orientia* that contains one species, *Orientiatsutsugamushi*, and *Rickettsia* that contains 28 species.

*Occidentia massiliensis* gen. nov., sp. nov. strain OS118^T^ is the type strain of *O. massiliensis* gen. nov., sp. nov., the type species of the new genus *Occidentia* gen. nov. within the family *Rickettsiaceae*. This strain, whose genome is described here, was isolated in France from the soft tick *Ornithodoros sonrai* collected in Senegal. *O. massiliensis* is an aerobic, rod-shaped, Gram-negative, obligate intracellular bacillus that may be cultivated in BME/CTVM2 cells.

Here we describe the features of *O. massiliensis*, together with the complete genomic sequencing and annotation. The 1,469,252 bp long genome (1 chromosome but no plasmid) contains 1,670 protein-coding and 41 RNA genes, including one rRNA operon.

## Introduction

*Occidentia massiliensis* gen. nov., sp. nov. strain OS18^T^ is the type strain of *O. massiliensis* gen. nov., sp. nov. This bacterium was isolated from an *Ornithodoros sonrai* tick collected in Senegal. It is an aerobic, rod-shaped, Gram-negative, obligate intracellular bacillus.

**Figure 1:**
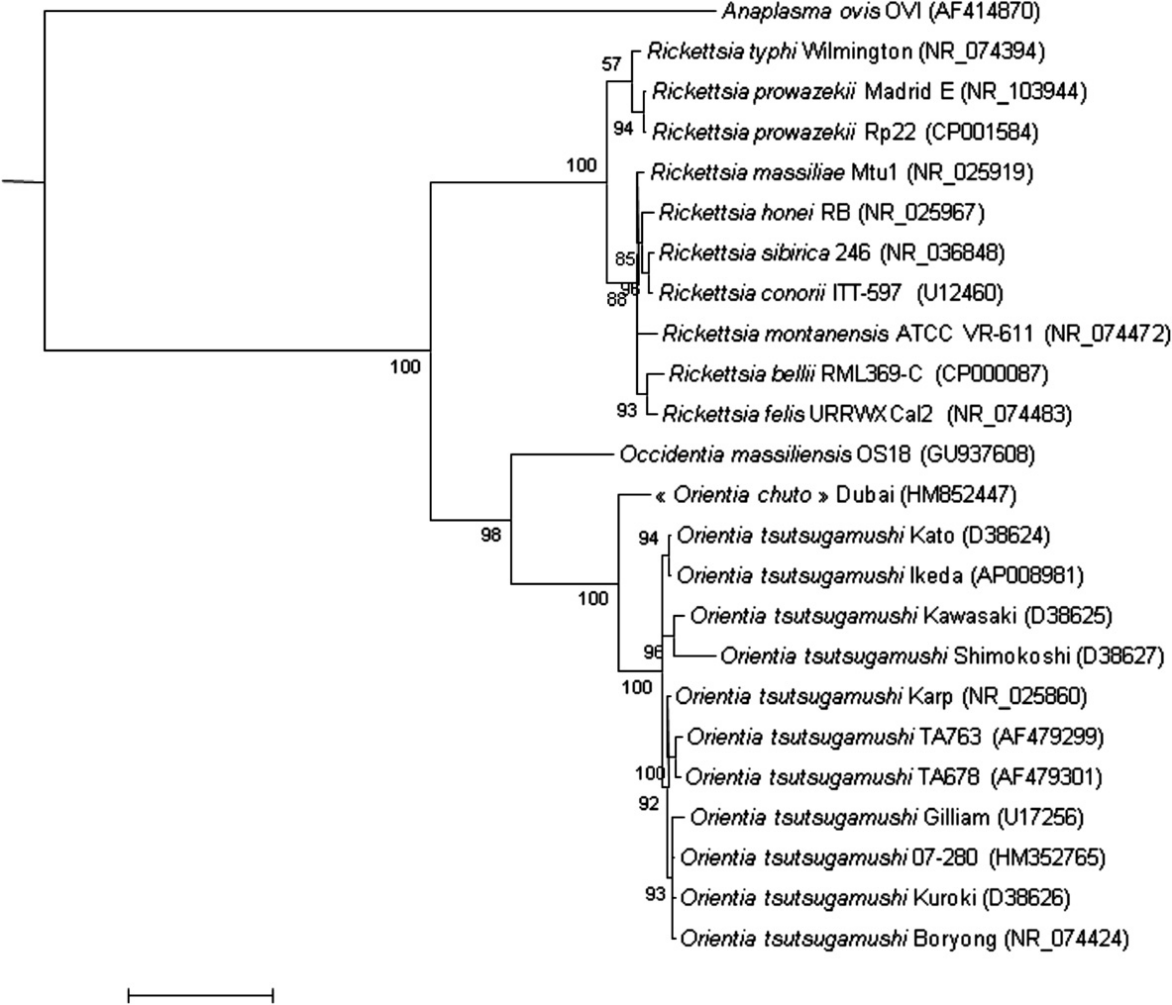
**A consensus phylogenetic tree showing the position of *Occidentia massiliensis *strain OS18**^**T **^**relative to other strains within the family *Rickettsiaceae*, based on 16S rDNA sequence comparison.** GenBank accession numbers are indicated in parentheses. Sequences were aligned using CLUSTALW, and phylogenetic inferences obtained using the Bayesian phylogenetic analysis [10] with the TOPALi 2.5 software (Biomathematics and Statistics Scotland, Edinburgh, UK) within the integrated MrBayes application [11], using the HKY85 + Г substitution model. Numbers at the nodes are percentages of bootstrap values from 100 replicates. *Anaplasma ovis* was used as the outgroup. The scale bar represents a 5% nucleotide sequence divergence.

The family *Rickettsiaceae* Pinkerton, [1] currently includes two genera: *Orientia* Tamura *et al.*[2] that contains one species, *Orientia tsutsugamushi* (Hayashi 1920, Tamura et al. 1995), and *Rickettsia* (da Rocha-Lima 1916) that contains 28 species [3]. Many members of this family have been detected and identified by PCR only, and have yet to be validly published [4,5]. The family *Rickettsiaceae* is composed of obligate intracellular bacteria that infect the cytoplasm and sometimes the nucleus of eukaryotic cells within which they live freely [6]. In addition, both *Rickettsia* species and *O. tsutsugamushi* are rod-shaped or coccoid, Gram-negative, bacteria intimately associated with arthropod hosts [7]. To date, none of the members of this family has been cultivated axenically. Many validly published species within the family *Rickettsiaceae* are pathogenic for humans and other vertebrates, causing spotted fevers or various forms of typhus. These diseases are transmitted by arthropods (mostly ticks, mites, lice or fleas). Other species of undescribed pathogenicity have only been detected in arthropods. Phylogenetically, *Occidentia massiliensis* gen. nov., sp. nov., is most closely related to *Orientia tsutsugamushi* (Figure 1) [2]. By comparison with *Rickettsia* species, *O. tsutsugamushi* differs in outer envelope layers [8], antigenic properties and by the absence of peptidoglycans and lipopolysaccharides. These phenotypic differences are supported by a 16S rRNA nucleotide sequence identity < 90.6%. In 2003, Fournier *et al*. developed genetic criteria to classify rickettsial isolates based on comparison of 16S rRNA and other genes [9]. The development of this strategy, combining sequences from several genes, notably housekeeping genes, was motivated by the small number of phenotypic criteria available for these strictly intracellular bacteria.

Here we present a summary classification and a set of features for *O. massiliensis* gen. nov., sp. nov., strain OS18^T^ (CSUR = P764, DSM = 24860) together with the description of the complete genomic sequencing and annotation. These characteristics support the circumscription of the genus *Occidentia* and its type species, *O. massiliensis* within the *Rickettsiaceae* family.

## Organism information

**Figure 2:**
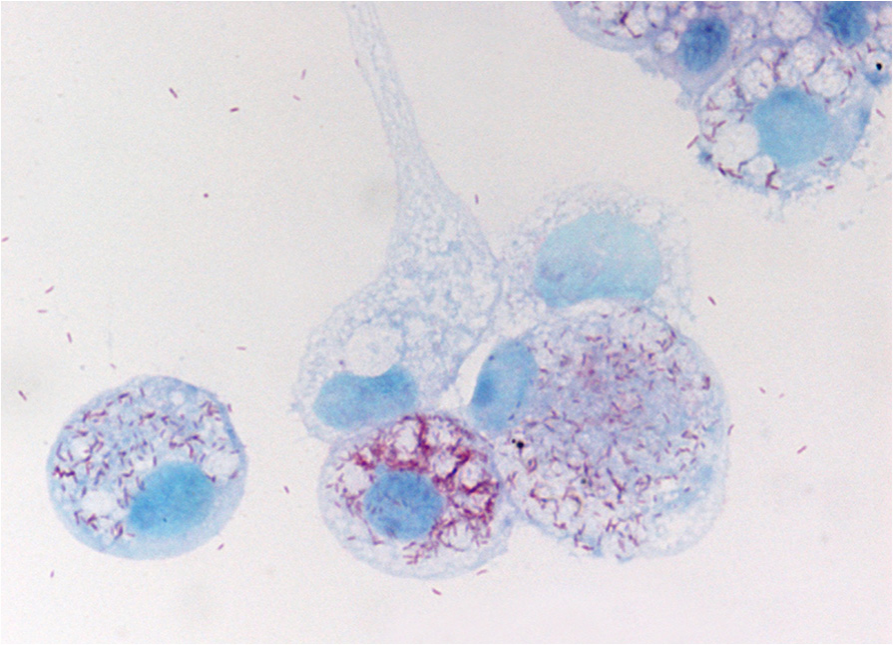
**Gimenez stain of *O. massiliensis *** **strain OS18**^**T **^**grown in BME/CTVM2 cells.**

In June 2009, 20 adult *Ornithodoros sonrai* soft ticks were collected from rodent burrows in the Soulkhou Thissé village (a rural village in the Guinean-Sudanian zone in Senegal, close to the city of Tambacounda) as part of a prospective study on tick-borne relapsing fever in West Africa. Ticks were kept alive until further processed. All ticks were disinfected, ground in Rinaldini solution and inoculated in a tick cell line monolayer (BME/CTVM2 cells from *Rhipicephalus microplus*) [12] using the shell-vial technique [13]. Shell vials were further incubated in an aerobic atmosphere at 28°C. Culture medium (Leibovitz-15 medium supplemented with 10% heat-inactivated fetal calf serum, 10% tryptose phosphate broth and 2 mM glutamine) was changed once a week. Strain OS18 (Table 1) was isolated in 2009 after one month of culture (Figure 2).

**Table 1:** **Classification and general features of *Occidentia massiliensis *** **strain OS18**^**T **^**according to the MIGS recommendations **[14]

**MIGS ID**	**Property**	**Term**	**Evidence code**^**a**^
	Current classification	Domain *Bacteria*	TAS [15]
		Phylum *Proteobacteria*	TAS [16]
		Class *Alphaproteobacteria*	TAS [17]
		Order *Rickettsiales*	TAS [6,18,19]
		Family *Rickettsiaceae*	TAS [1,18,19]
		Genus *Occidentia*	IDA
		Species *Occidentia massiliensis*	IDA
		Type strain OS18^T^	IDA
	Gram stain	Negative	IDA
	Cell shape	Rod	IDA
	Motility	Unknown	IDA
	Sporulation	Nonsporulating	IDA
	Temperature range	Mesophilic	IDA
	Optimum temperature	28°C	IDA
MIGS-6.3	Salinity	Unknown	IDA
MIGS-22	Oxygen requirement	Aerobic	IDA
	Carbon source	Unknown	NAS
	Energy source	Unknown	NAS
MIGS-6	Habitat	*Ornithodoros sonrai*	IDA
MIGS-15	Biotic relationship	Obligate intracellular	IDA
	Pathogenicity	Unknown	
	Biosafety level	2	
MIGS-14	Isolation	*Ornithodoros sonrai*	IDA
MIGS-4	Geographic location	Senegal	IDA
MIGS-5	Sample collection time	June 2009	IDA
MIGS-4.1	Latitude	14.05	IDA
MIGS-4.2	Longitude	−15.516667	IDA
MIGS-4.3	Depth	0.5 m below surface	IDA
MIGS-4.4	Altitude	45 m above sea level	IDA

^a^Evidence codes - IDA: Inferred from Direct Assay; TAS: Traceable Author Statement (*i.e*., a direct report exists in the literature); NAS: Non-traceable Author Statement (*i.e*., not directly observed for the living, isolated sample but based on a generally accepted property for the species or anecdotal evidence). Evidence codes come from the Gene Ontology project [20]. If the evidence is IDA, then the property was directly observed for a live isolate by one of the authors or an expert mentioned in the acknowledgements.

Five other morphologically and genetically indistinguishable isolates were recovered from five other *O. sonrai* ticks from the same batch. The 16S rRNA nucleotide sequence (GenBank accession number GU937608) of *Occidentia massiliensis* strain OS18T was 93.7% similar to *Orientia tsutsugamushi* strain Gilliam (GenBank accession number D38622), the phylogenetically closest species, but formed a separate, well-supported (bootstrap value 98%) sister branch to the *O. tsutsugamushi* species (Figure 1). This value was lower than the 95% 16S rRNA gene sequence threshold recommended by Stackebrandt and Elbers to delineate a new genus without carrying out DNA-DNA hybridization [21].

**Figure 3:**
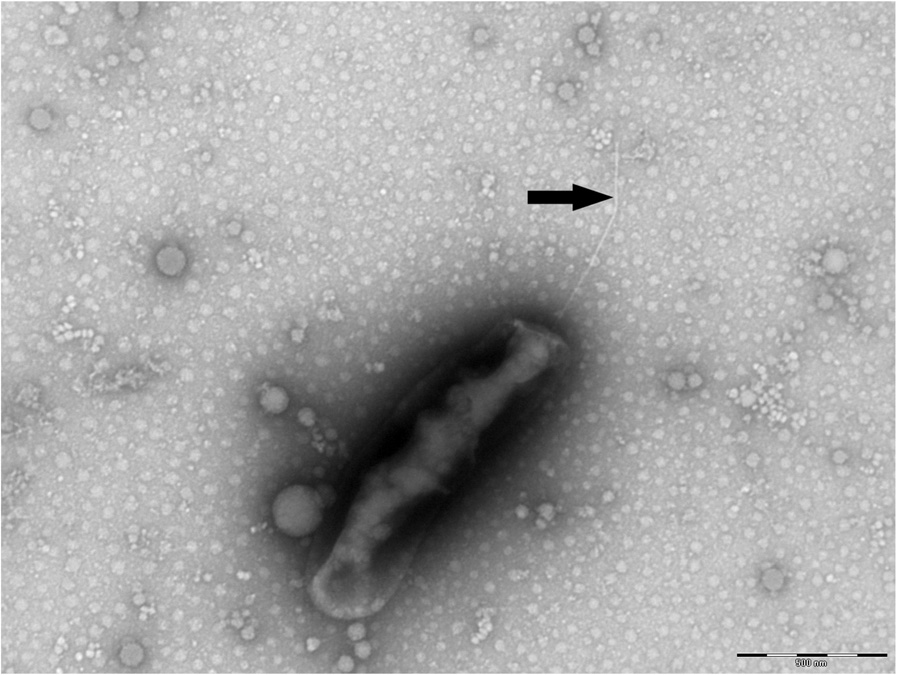
**Scanning electron micrograph of *O. massiliensis *strain OS18**^**T**^**, made using a Morgagni 268D (Philips) transmission electron microscope at an operating voltage of 60 kV.** The arrow shows a monotrichous flagellum. The scale bar represents 500 nm.

**Figure 4:**
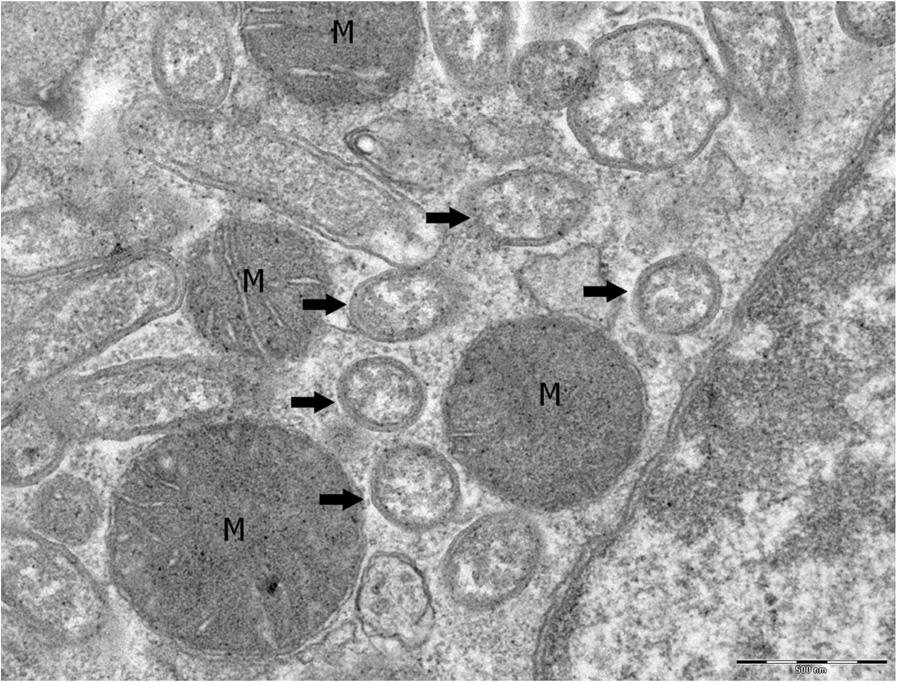
**Transmission electron micrograph of *O. massiliensis *strain OS18**^**T **^**grown in BME/CTVM2 cells made using uranyl acetate staining and a Morgagni 268D (Philips) at an operating voltage of 60 kV.** The bacteria (arrows) characteristically surround mitochondria (M). The scale bar represents 500 nm.

**Figure 5:**
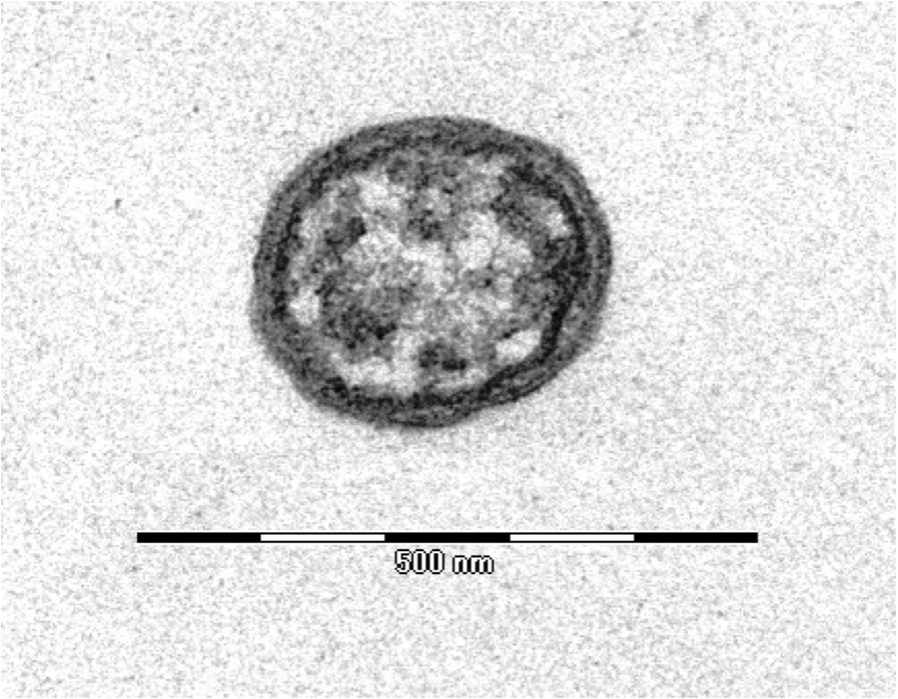
**Transmission electron micrograph of a single *O. massiliensis *** **strain OS18**^**T **^**cell made using uranyl acetate staining and a Morgagni 268D (Philips) at an operating voltage of 60 kV. The scale bar represents 500 nm.**

Growth was attempted at 28°C in an aerobic atmosphere, which were the culture conditions required for the BME/CTVM2 cell line, and at 37°C in an aerobic atmosphere in L929 and XTC cell lines. Bacteria grew in BME/CTVM2 cells but no growth was obtained in L929 and XTC cell lines. Bacterial cells grown inside BME/CTVM2 cells were Gimenez-positive but weakly Gram-negative. Scanning electron microscopy revealed that cells were rod-shaped with one “rounded” end and another “blunt” end (Figure 3). A monotrichous flagellum was attached to the “blunt” end. Cells had a mean length and width of 1.23 ± 0.19 μm and 0.42 ± 0.06 μm, respectively. Bacteria were abundant within the cytoplasm but not the nucleus of tick cells (Figure 2). Typically, the highest concentration of bacteria was seen around mitochondria within cells (Figure 4). Contrary to *Rickettsia* species and *O. tsutsugamushi*, we did not identify evident differences between the inner and outer leaflets of the cell wall (Figure 5), although the periplasmic space was unusually large (0.028 ± 0.007 μm).

### Genome sequencing information

#### Genome project history

The organism was selected for sequencing on the basis of its phylogenetic position and 16S rRNA similarity to members of the family *Rickettsiaceae*. Nucleotide sequence similarity levels of these genes suggested that strain OS18^T^ represents a new genus within the family *Rickettsiaceae*. It was the first genome of *Occidentia massiliensis* gen. nov., sp. nov. The Genbank accession number is CANJ00000000 and consists of 47 large contigs (>1.5 kb) in 18 scaffolds. Table 2 shows the project information and its association with MIGS version 2.0 compliance [14].

**Table 2:** Project information

**MIGS ID**	**Property**	**Term**
MIGS-31	Finishing quality	High-quality draft
MIGS-28	Libraries used	One paired-end 3-kb library
MIGS-29	Sequencing platforms	454 GS FLX Titanium
MIGS-31.2	Fold coverage	23.2×
MIGS-30	Assemblers	Newbler version 2.5.3
MIGS-32	Gene calling method	Prodigal
	GenBank ID	CANJ00000000
	GenBank Date of Release	April 15, 2013
MIGS-13	Project relevance	Biodiversity of the *Ornithodoros sonrai* tick microbial flora

#### Growth conditions and DNA isolation

*O. massiliensis* gen. nov., sp. nov., strain OS18^T^ (CSUR = P764, DSM = 24860) was grown aerobically in BME/CTVM2 cell line at 28°C. Infected cells were harvested from 20 culture flasks. Bacterial purification using a renografin gradient was performed as previously described [22]. A total of 200 μL of bacterial suspension was diluted in 1 ml TE buffer for lysis treatment. After incubation with 2.5 μg/μL lysozyme for 30 minutes at 37°C, the lysis was performed with 1% laurylsarcosyl and 50 μg/μL RNAse A for 1 hr at 37°C, followed by an overnight incubation at 37°C with proteinase K. The DNA was purified three times by phenol-chloroform extraction and then precipitated by addition of ethanol at −20°C overnight. After centrifugation, the DNA was resuspended in 199 μL TE buffer. The DNA concentration was measured by the Quant-it Picogreen kit (Invitrogen) on the Genios-Tecan fluorometer at 69.12 ng/μl.

### Genome sequencing and assembly

A 3 kb paired-end sequencing strategy (454 GS FLX Titanium, Roche) was selected. DNA (5 μg) was mechanically fragmented on the Covaris device (KBioScience-LGC Genomics, Teddington, UK) through miniTube-Red 5 kb with an enrichment size of 3–4 kb. The DNA fragmentation was visualized using the Agilent 2100 BioAnalyzer on a DNA labchip 7500 with an average size of 3.2 kb. Circularization and nebulization were performed and generated a pattern with an average size of 580 bp. After PCR amplification over 17 cycles followed by double size selection, the single-stranded paired-end library was then quantified on the Genios-Tecan fluorometer with the Quant-iT ribogreen (Invitrogen) at 1,120 pg/μL. The library concentration equivalence was calculated as 3.55 × 10^9^ molecules/μL. The library was stored at −20°C until further use.

The library was clonally amplified with 0.7 cpb in 4 emPCR reactions, with the GS Titanium SV emPCR Kit (Lib-L) v2 (Roche). The yield was calculated at 9.16%, within the recommended yield range of between 5 and 20% from the Roche procedure. After amplification, 790,000 beads from the emPCR reaction were loaded on a ¼ region on the GS Titanium PicoTiterPlate PTP Kit 70 × 75 and sequenced with the GS FLX Titanium Sequencing Kit XLR70 (Roche). The run was analyzed on the cluster through the gsRunBrowser and Newbler assembler (Roche). A total of 103,355 passed filter wells were obtained and generated 34.1 Mb of DNA sequence with an average read length of 330 bp.

The passed filter sequences were assembled using Newbler with 90% identity and 40 bp for overlap requirements. The final assembly identified 18 scaffolds and 47 large contigs (>1.5 kb) generating a genome size of 1,47 Mb which corresponds to a coverage of 23.2×.

### Genome annotation

Open reading frames were predicted using PRODIGAL with default parameters [23], but predicted ORFs were excluded if they spanned a sequencing gap region. The functional annotation of protein sequences was performed using BLASTP against the GenBank and Clusters of Orthologous Groups (COG) databases [24]. The prediction of tRNAs and rRNAs was carried out using the tRNAScan-SE [25] and RNAmmer [26] tools, respectively. Lipoprotein signal peptides and numbers of transmembrane helices were predicted using SignalP [27] and TMHMM [28], respectively. ORFans were identified if their BLASTP *E*-value was lower than 1e-03 for alignment length greater than 80 amino acids. If alignment lengths were smaller than 80 amino acids, we used an *E*-value of 1e-05. Such parameter thresholds have already been used in previous works to define ORFans. To estimate the mean level of nucleotide sequence similarity at the genome level between *O. massiliensis* and another 4 members of the family *Rickettsiaceae* (Table 3), we used the Average Genomic Identity Of gene Sequences (AGIOS) home-made software [29]. Briefly, this software combines the Proteinortho software [30] for detecting orthologous proteins in pairwise comparisons of genomes, then retrieves the corresponding genes and determines the mean percentage of nucleotide sequence identity among orthologous ORFs using the Needleman-Wunsch global alignment algorithm.

**Table 3:** Nucleotide content and gene count levels of the genome

**Attribute**	**Value**	**% of total**^**a**^
Size (bp)	1,469,252	100
DNA G + C content (bp)	426,780	29.05
Total genes	1,670	100
RNA genes	41	2.66
Protein-coding genes	1,502	97.34
Protein with predicted function (COGs + NR)	1,099	73.17
Genes assigned to COG	1,062	70.71
Genes with peptide signal	131	8.72
Genes with transmembrane helices (≥3)	331	22.04

^a^The total is based on either the size of the genome in base pairs or the total number of protein coding genes in the annotated genome.

## Genome properties

The genome is 1,469,252 bp long (one chromosome, no plasmid) with a 29.05% GC content (Table 4). It is composed of 301 contigs (18 scaffolds). Of the 1,543 predicted genes, 1,502 were protein-coding genes, and 41 were RNAs (1 rRNA operon and 38 tRNA genes).

**Table 4:** Number of genes associated with the 25 general COG functional categories

**Code**	**Value**	**%age**	**Description**
J	158	10.52	Translation
A	0	0	RNA processing and modification
K	40	2.66	Transcription
L	107	7.12	Replication, recombination and repair
B	0	0	Chromatin structure and dynamics
D	20	1.33	Cell cycle control, mitosis and meiosis
Y	0	0	Nuclear structure
V	17	1.13	Defense mechanisms
T	32	2.13	Signal transduction mechanisms
M	114	7.59	Cell wall/membrane biogenesis
N	2	0.13	Cell motility
Z	0	0	Cytoskeleton
W	0	0	Extracellular structures
U	64	4.26	Intracellular trafficking and secretion
O	72	4.79	Posttranslational modification, protein turnover and chaperones
C	94	6.26	Energy production and conversion
G	45	3	Carbohydrate transport and metabolism
E	90	5.99	Amino acid transport and metabolism
F	20	1.33	Nucleotide transport and metabolism
H	40	2.66	Coenzyme transport and metabolism
I	29	1.93	Lipid transport and metabolism
P	52	3.46	Inorganic ion transport and metabolism
Q	14	0.93	Secondary metabolites biosynthesis, transport and catabolism
R	146	9.72	General function prediction only
S	53	3.52	Function unknown
X	440	29.29	Not in COGs

The total is based on the total number of protein-coding genes in the annotated genome.

**Figure 6:**
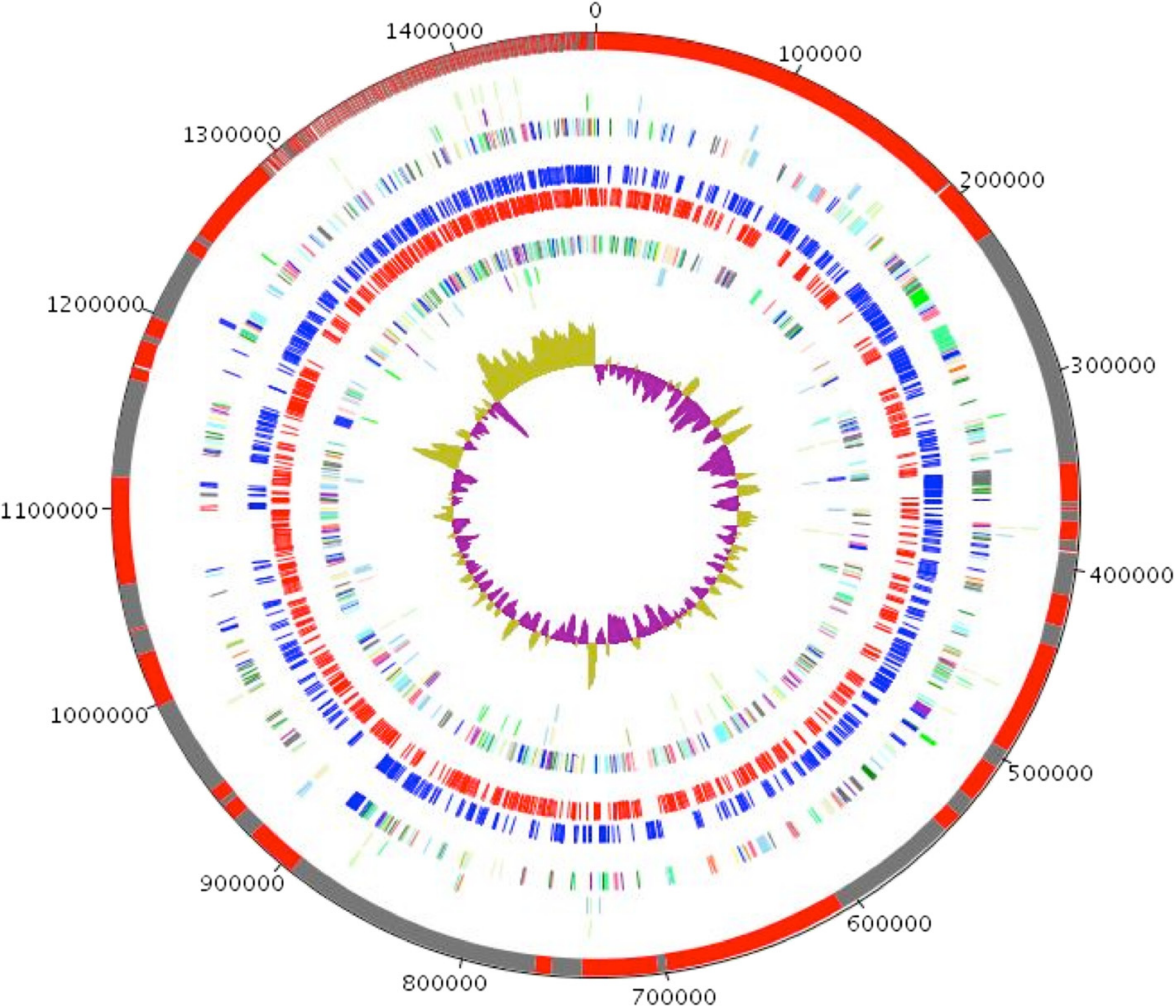
**Graphical circular map of the chromosome.** From outside in: contigs (red / grey), COG category of genes on the forward strand (three circles), genes on forward strand (blue circle), genes on the reverse strand (red circle) and COG category of genes on the reverse strand (three circles). The inner-most circle shows the G + C skew, with purple and olive indicating negative and positive values, respectively.

A total of 1,099 genes (73.17%) were assigned a putative function (by COG or by NR blast), and 185 genes were identified as ORFans (12.32%). The remaining genes were annotated as hypothetical proteins (165 genes = > 10.99%). The distribution of genes into COGs functional categories is presented in Table 5 and Figure 6. The properties and the statistics of the genome are summarized in Tables 4 and 5.

**Table 5:** Bacterial genomes used for the genomic comparison

**Species**	**Numberof proteins**	**Genome Size (Mb)**	**G + C content**
*O. massiliensis* strain OS18^T^	1502	1.47	29.0
*O. tsutsugamushi* strain Boryong	1182	2.13	30.5
*O. tsutsugamushi* strain Ikeda	1967	2.01	30.5
*R. bellii* strain RML369-C	1428	1.52	31.6
*R. prowazekii* strain Madrid E	842	1.1	29.0

### Genomic comparison of *O. massiliensis* and other members of the family *Rickettsiaceae*

We compared the genome sequence of *O. massiliensis* strain OS18^T^ to those of *O. tsutsugamushi* strains Boryong (GenBank accession number NC_009488) and Ikeda (NC_010793), and *R. prowazekii* strain Madrid E (NC_000963) and *R. bellii* strain RML369-C (NC_007940). *Occidentia massiliensis* strain OS18^T^ had a much smaller genome (1,469,252 bp, 2,127,051 bp and 2,008,987 bp, respectively), fewer genes (1,670, 2,216 and 2,005 genes, respectively) and a lower G + C content (29.05%, 30.5%, 30.5% than *O. tsutsugamushi* strains Boryong and Ikeda (Table 6). However, when compared to *Rickettsia* species, *O. massiliensis* had a larger genome than *R. prowazekii* (1,469,252 bp and, 1,111,523 bp, respectively), but smaller than *R. bellii* (1,522,076 bp). In contrast, the G + C content of *O. massiliensis* was identical to that of *R. prowazekii* (29%) but lower than *R. bellii* (31.6%). In addition, *O. massiliensis* exhibited AGIOS values of 73.58 and 73.62% when compared to *O. tsutsugamushi* strains Boryong and Ikeda, respectively, higher than those obtained by comparison with *R. bellii* and *R. prowazekii* (68.7 and 69.45%, respectively, Table 3). However, these values were lower than those obtained between *O. tsutsugamushi* strains (97.49%) and *Rickettsia* species (81.57%), but similar to those obtained by comparison of *Orientia* and *Rickettsia* genomes (67.48 to 68.08%, Table 3), thus confirming the new genus status of *O. massiliensis*.

**Table 6:** **Genomic Comparison of *O. massiliensis *** **strain OS18**^**T **^**with other members of the family *Rickettsiaceae***

**Species**	***O. massiliensis***	***O. tsutsugamushi *** **Boryong**	***O. tsutsugamushi *** **Ikeda**	***R. bellii***	***R. prowazekii***
*O. massiliensis*	**1,502**	73.58	73.62	68.70	69.45
*O. tsutsugamushi* Boryong	564	**1,182**	97.49	67.48	68.06
*O. tsutsugamushi* Ikeda	572	592	**1,967**	67.58	68.08
*R. bellii*	603	502	507	**1,428**	81.57
*R. prowazekii*	588	482	489	598	**842**

Upper right, AGIOS values; lower left, number of orthologous proteins; bold number indicate the numbers of protein-coding genes.

## Conclusions

Strain OS18 shares a maximum 93.76% 16S rRNA identity with *O. tsutsugamushi*, its closest phylogenetic neighbor, and 91.61% with *R. prowazekii*. These values are lower than the 95% threshold proposed by Stackebrandt and Ebers to delineate genera [21]. In addition, the genomic comparison of *O. massiliensis* and members of two genera from the family *Rickettsiaceae* demonstrated that the former species exhibits AGIOS values similar to those obtained by comparison of genera, but much lower than those obtained by intra-genus strain comparison.

Moreover, the morphological (monotrichous flagellum, weak coloration by Gimenez staining, concentration around the mitochondria inside infected cell, large periplasmatic space) and epidemiological (association with soft ticks, inability to grow in L929 and XTC cell lines) evidence also differentiates strain OS18 from other members of the family *Rickettsiaceae*.

On the basis of phenotypic, phylogenetic and genomic analyses, we formally propose the creation of *Occidentia massiliensis* gen. nov., sp. nov., that contains strain OS18^T^. This bacterium has been isolated in France from a tick collected in Senegal.

### Description of *Occidentia* gen. nov

*Occidentia* (oc.ci.den’tia N.L. fem. Adj. *occidentia*, of the occident, for the western part of Africa where the tick from which the type strain was isolated, was collected, and in contrast with *Orientia*, the name of its phylogenetically closest relative, distributed in Asia).

Gimenez positive and weakly Gram-negative rods. Strictly intracellular. Non-spore-forming. Grows in BME/CTVM2 tick cells at 28°C. The bacteria multiply freely in the cytoplasm, but not the nucleus, of host cells. Monotrichous flagellum. Habitat: *Ornithodoros sonrai*. Type species: *Occidentia massiliensis*.

### Description of *Occidentia massiliensis* gen. nov., sp. nov.

*Occidentia massiliensis* (mas.si.li.en’sis. L. gen. fem. n. massiliensis, of *Massilia*, the Latin name of Marseille, France, where strain OS18^T^ was first grown, identified and characterized).

Gimenez positive and weakly Gram-negative rods. Strictly intracellular. Non-spore-forming. Grows in BME/CTVM2 tick cells at 28°C. The bacteria multiply freely in the cytoplasm, but not the nucleus, of host cells. Monotrichous flagellum. The mean length and width of the bacteria are 1.23 ± 0.19 μm and 0.42 ± 0.06 μm, respectively. Bacteria exhibit a large periplasmic space of 0.028 ± 0.007 μm. The genome is 1,469,252-bp long and contains 1,502 protein-coding and 41 RNA genes. The 16S rRNA and genomic sequences are deposited in GenBank under accession numbers GU937608 and CANJ00000000, respectively. The genomic G + C content is 29.05%. The type strain OS18^T^ (CSUR = P764, DSM = 24860) was isolated from an *Ornithodoros sonrai* soft tick collected in Senegal.

## Competing interests

The authors declare that they have no competing interests.

## Authors’ contribution

OM isolated the bacterium, performed the photographies and electron micrography, phylogenetic analysis and drafted the manuscript, TTN carried out the genome sequencing, LBS participated in the cell culture, RP performed the genome comparison, PEF supervised the genomic studies and drafted the manuscript, DR initiated and organised the study and drafted the manuscript.
